# Vericiguat for Heart Failure with Reduced Ejection Fraction

**DOI:** 10.1007/s11886-021-01580-6

**Published:** 2021-08-19

**Authors:** Carlo Mario Lombardi, Giuliana Cimino, Matteo Pagnesi, Andrea Dell’Aquila, Daniela Tomasoni, Alice Ravera, Riccardo Inciardi, Valentina Carubelli, Enrico Vizzardi, Savina Nodari, Michele Emdin, Alberto Aimo

**Affiliations:** 1 Cardiology; ASST Spedali Civili di Brescia and Department of Medical and Surgical Specialties, Radiological Sciences and Public Health, University of Brescia, P.zza Spedali Civili 1, 25123 Brescia, Italy; 2 Institute of Life Sciences, Scuola Superiore Sant’Anna, Piazza Martiri della Libertà 33, 56124 Pisa, Italy; 3 Cardiology Division, University Hospital of Pisa, Piazza Martiri della Libertà 33, 56124 Pisa, Italy; 4 Cardiology Department, Fondazione Toscana Gabriele Monasterio, Pisa, Italy

**Keywords:** Heart failure, Soluble guanylate cyclase, Cyclic guanosine monophosphate, Treatment, Pathophysiology, Vericiguat

## Abstract

**Purpose of Review:**

The nitric oxide (NO)-soluble guanylate cyclase (sGC)-cyclic guanosine monophosphate (cGMP) pathway plays an important role in the regulation of cardiovascular function, and it is disrupted in heart failure (HF), resulting in decreased protection against myocardial injury. Impaired NO-sGC-cGMP signaling in HF is secondary to reduced NO bioavailability and altered redox state of sGC, which becomes less responsive to NO. The sGC activator cinaciguat increases cGMP levels by direct NO-independent activation of sGC and may be particularly effective in conditions of increased oxidative stress and endothelial dysfunction, and therefore reduced NO levels, at the expense of a greater risk of hypotension. Conversely, sGC stimulators (riociguat and vericiguat) enhance sGC sensitivity to endogenous NO, thus exerting a more physiological action.

**Recent Findings:**

Clinical trials have suggested the benefit of vericiguat in patients with high-risk HF; in particular, a lower incidence of death from cardiovascular causes or HF hospitalization.

**Summary:**

Adding vericiguat may be considered in individual patients with HF, and reduced left ventricular ejection fraction (HFrEF) particularly those at higher risk of HF hospitalization.

## Introduction

The burden of heart failure (HF) is increasing progressively due to population aging and better prognosis of patients with acute cardiovascular events [1]. Over 20 million people worldwide are currently affected by HF [2]. Despite recent advances in HF management, patients with symptomatic HF still have a poor prognosis [3]. Community-based studies indicate that up to 40% of patients die within 1 year from diagnosis and 60–70% within 5 years, mainly from worsening HF or sudden cardiac death (SCD) [4, 5].

**Fig. 1:**
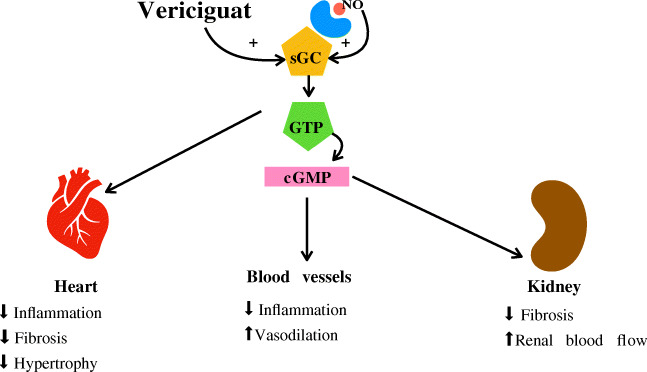
Cardiovascular and noncardiovascular effects of vericiguat. Figure legend: NO, nitroxigen oxide; sGC, soluble guanylate cyclase; GTP, guanosine triphosphate; cGMP, cyclic guanosine monophosphate

Despite the efficacy of the standard of care (SOC) therapy for heart failure with reduced ejection fraction (HFrEF), namely beta-blockers, angiotensin-converting enzyme inhibitors (ACEi), angiotensin receptor blockers (ARBs), mineralocorticoid receptor antagonists (MRA), and device therapies [6, 7], there are still unmet needs and new opportunities for advancements in care (Figure 1). Treatment with the combined ARB/neprilysin inhibitor *sacubitril/valsartan* has been associated with lower rates of hospital admissions and mortality from HF, and a greater likelihood of recovery from LV dysfunction (reverse remodeling) [8].

The major evidence on the efficacy of sacubitril/valsartan came from the PARADIGM-HF study, a pivotal phase III, randomized, double-blind study comparing sacubitril/valsartan and enalapril, conducted in 8442 patients with chronic HF class NYHA II–IV and with LVEF ≤35% [9]. The study, statistically sized for the evaluation of the reduction of cardiovascular death, was stopped early due to the superiority of treatment with sacubitril/valsartan compared to that assigned to enalapril. In fact, it reduced the primary composite risk endpoint of cardiovascular death or first hospitalization for HF by 20%, an effect that was observed early and was maintained throughout the duration of the study. Furthermore, the favorable effects of sacubitril/valsartan compared to ACEs were observed in the different phases of the HF, from the earliest to the later.

In view of these promising results, it was considered to evaluate the effect of early therapy initiation. In this regard, the PIONEER-HF trial, a double-blind, randomized, multicenter study, compared sacubitril/valsartan and enalapril in a population of 882 patients hospitalized for an episode of heart failure and with elevated levels of NT-proBNP. The results of the study showed that the initiation of therapy with sacubitril/valsartan, following hemodynamic stabilization, leads to a significant reduction in NT-proBNP levels at 8 weeks compared to enalapril [10].

Among the novel therapeutic options, *sodium-glucose co-transporter 2 inhibitors* (SGLT2i) decrease the risk of HF hospitalizations in patients with type 2 diabetes [11]. The benefits of SGLT2i on HF seemed greater than expected based on the diuretic and hypoglycemic effects of these drugs. These observations have raised the possibility that SGLT2i could reduce morbidity and mortality in patients with established HF, including those without diabetes [12]. Indeed, the DAPA-HF trial showed that dapagliflozin reduced the risk of the composite of cardiovascular death or HF hospitalization by 26% over the SOC, and also the individual components of the composite endpoint. Additionally, the study showed a significant improvement in patient-reported quality of life, as measured by the Kansas City Cardiomyopathy Questionnaire (KCCQ) symptom score, and a significant reduction in all-cause mortality by 17% in patients on dapagliflozin [13]. Recently, the EMPEROR-Reduced trial demonstrated that empagliflozin reduces the risk of cardiovascular death and HF hospitalization in patients with moderate-to-severe HFrEF with or without diabetes, and slows the deterioration of renal function [14]. In a recent study on patients with diabetes and a recent HF decompensation, sotagliflozin resulted in a significantly lower total number of deaths from cardiovascular causes and HF hospitalizations compared with placebo, despite premature trial discontinuation [15]

*Omecamtiv mecarbil* (OM), a first-in-class selective cardiac myosin activator, improved cardiac function and decreased left ventricular (LV) volumes, heart rate, and N-terminal pro-B-type natriuretic peptide (NT-proBNP) in patients with chronic HF. In the *GALACTIC*-*HF* trial, treatment with OM achieved the primary composite efficacy endpoint of cardiovascular death or HF events (HF hospitalization and other urgent treatment for HF) compared with placebo in patients with HFrEF on SOC. No significant reduction in the secondary endpoint of cardiovascular death was observed. Rates of adverse events, including major ischemic cardiac events, were well balanced between treatment arms [16, 17].

## Modulation of sGC Cyclase Activity

Preclinical and clinical evidence supports the potentiation of the nitric oxide (NO)-soluble guanylate cyclase (sGC)-cyclic guanosine monophosphate (cGMP) pathway as a potential therapeutic target in HF. Under physiologic conditions, the cGMP pathway is a critical regulator of myocardial energetics, cardiac performance, and endothelial function [18–21]. In HF, increased inflammation and vascular dysfunction result in lower NO bioavailability, leading to a reduced in cGMP synthesis [21]. cGMP deficiency causes systemic, coronary, and renal microcirculatory dysfunction, which may lead to progressive myocardial damage and further inflammation [18, 19, 22]. Deranged cGMP signaling has been correlated with worse clinical outcomes among patients with HFrEF [18].

sGC activators, such as cinaciguat, increases cGMP levels by direct, NO-independent activation of sGC, and may be particularly effective in conditions of increased oxidative stress and endothelial dysfunction, and therefore reduced NO levels, but this comes at the expense of a greater risk of hypotension. Conversely, sGC stimulators (riociguat and vericiguat), by enhancing sGC sensitivity to endogenous NO, exert a more physiological action, possibly explaining their neutral effects on blood pressure.

The availability of oral sGC stimulators has provided the opportunity to test the hypothesis that enhancement of the cGMP pathway by a sGC stimulator will favorably influence the natural history of chronic HFrEF. The survival benefit demonstrated for vericiguat (see below) could derive from a potentiation of the effects of natriuretic peptides on the kidneys, and then the induction of diuresis and natriuresis, rather than decreased cardiac afterload (5).

## Vericiguat in HFrEF

Experimental studies have suggested multiple potential benefits of sGC stimulators including prevention, or even reversal, of LV hypertrophy and fibrosis, as well as reduction of LV afterload due to systemic and pulmonary vasodilation [23]. Cardiovascular and noncardiovascular effects of vericiguat are summarized in Table 1. Therefore, restoration of an adequate NO-sGC–cGMP signaling has been proposed as an important treatment target in HF. Vericiguat enhances sGC sensitivity to endogenous NO and has been studied in a phase 2 (SOCRATES-REDUCED) and a phase 3 trial of patients with HFrEF (VICTORIA).

**Table 1:** Clinical trials on vericiguat in heart failure with reduced ejection fraction

Trial	Inclusion criteria	Patients	Treatment arm	Results	Safety
SOCRATES-REDUCED^21^	HF, LVEF <45%, <4 weeks from HF decompensation	351	Vericiguat (1.25 mg, 2.5 mg, 5 mg, 10 mg daily) for 12 weeks vs. placebo	Pooled vericiguat vs. placebo: no significant difference in Δlog(NT-proBNP) from baseline to week 12 (p=0.15).	Any adverse event: 71.4% vericiguat 10 mg, 77.2% placebo
VICTORIA^23^	HF, NYHA II-IV, LVEF <45%, BNP ≥300 ng/L (≥500 ng/L if AF) or NT-proBNP ≥1000 ng/L (≥1600 ng/L if AF), HF hospitalization <6 months or worsening HF requiring iv diuretics <3 months	5050	Vericiguat (target dose 10 mg daily) vs. placebo	Primary endpoint (CV death or HF hospitalization): HR 0.90 (0.82–0.98) HF hospitalization: HR 0.90 (0.81–1.00) Death or HF hospitalization: HR 0.90 (0.83–0.98)	Symptomatic hypotension: 9.1% vericiguat vs. 7.9% placebo (*p*=0.12). Syncope: 4.0% vs. 3.5% (p=0.30) Anemia: 7.6% vs. 5.7% (serious AE in 1.6% vs. 0.9%)

*AE*, adverse event; *AF*, atrial fibrillation; *BNP*, B-type natriuretic peptide; *CV*, cardiovascular; *HF*, heart failure; *LVEF*, left ventricular ejection fraction; *NT-proBNP*, N-terminal pro-B-type natriuretic peptide; *NYHA*, New York Heart Association

SOCRATES-REDUCED enrolled 456 patients with LVEF less than 45% and a recent episode of HF decompensation, defined by worsening HF symptoms requiring hospitalization or outpatient administration of intravenous diuretics, signs of congestion, and elevated natriuretic peptide level, excluding those with estimated glomerular filtration rate (eGFR) <30 mL/min/1.73 m^2^ and systolic blood pressure <110 or ≥160 mmHg. Patients were randomized to 5 arms (target maximal doses of 1.25 mg, 2.5 mg, 5 mg, and 10 mg once daily or placebo). Only 77% of patients completed the 12-week follow-up, and 72% of patients randomized to the highest dose reached the target 10 mg dose. The change in log-transformed NT-proBNP over 12 weeks did not differ significantly in the pooled vericiguat group and the placebo arm, while the exploratory comparison between vericiguat 10 mg and placebo achieved statistical significance (*p*=0.048). Patients on the highest vericiguat dose displayed also a greater increase in LVEF (*p*=0.02). Vericiguat therapy did not seem to affect hemodynamic function and appeared safe, with lower rates of serious adverse events than placebo [24•].

The phase 3 VICTORIA trial enrolled 4872 subjects with HFrEF and a history of HF decompensation requiring hospitalization over the last 6 months and/or intravenous diuretics <3 months and elevated circulating NT-proBNP. It examined the efficacy and safety of vericiguat compared with placebo on the background of SOC. Patients were randomized to placebo or vericiguat 2.5 mg once daily, up-titrated to 5 mg and then to 10 mg at 2-week intervals. Over a 10.8-month median follow-up, a high rate of events was observed, with nearly 20% (primary endpoint) at 4 months, as compared to 5% in DAPA-HF [25]. Patients on vericiguat had a lower incidence of the primary endpoint of cardiovascular death or first HF hospitalization (hazard ratio [HR] 0.90, 95% confidence interval [CI] 0.82–0.98; *p*=0.02), with a number needed to treat of 24. This result was driven by a lower incidence of first HF hospitalization (HR 0.90, 95% CI 0.81–1.00). Vericiguat seemed less effective in patients in the highest quartile of NT-proBNP (>5314 ng/L), those aged ≥75 years, with eGFR 15–30 mL/min/1.73 m^2^, or with LVEF 40–45%, although *p* values for interaction were not provided, and the trial was underpowered for subgroup analyses. The rates of symptomatic hypotension or syncope did not differ significantly between patients on vericiguat or placebo.

Serious adverse events occurred in 32.8% of the patients in the vericiguat group and in 34.8% of the patients in the placebo group. Adverse events (serious and nonserious) occurred in 80.5% of the patients receiving vericiguat and in 81.0% of the patients receiving placebo. No serious adverse events of vericiguat on electrolytes or renal function emerged. Drug tolerability was further confirmed by the high rate of target dose achievement (89%) [26••].

A VICTORIA substudy demonstrated a significant benefit of vericiguat compared to placebo on cardiovascular death and HF hospitalization in patients with baseline NT-proBNP levels up to 8000 ng/L, further amplified in case of NT-proBNP levels up to 4000 ng/L [27]. Furthermore, another subanalysis showed that the benefit of vericiguat did not differ significantly across 3 subgroups identified by the recent HF-related event (HF hospitalization in the previous 3 months, HF hospitalization in the previous 3–6 months, outpatient intravenous diuretics for worsening HF in the previous 3 months) [28].

Finally, the VICTORIA trial photographed the results obtainable with vericiguat and optimal medical therapy in a very complex population. It is no coincidence that the incidence of the primary composite outcome occurred in a high percentage of cases (33.6%) and the separation of the survival curves was observed early, after approximately 3 months, when an event rate was recorded in the placebo group with a surprisingly high optimal therapy rate. Indeed, NYHA class and baseline NT-proBNP were statistically higher in patients enrolled in VICTORIA compared to trials such as PARADIGM-HF and DAPA-HF [9, 25]. In addition, the study protocol required patient enrollment within 6 months of hospitalization for HF or, without hospitalization, within 3 months of IV diuretic therapy; OMT with > 90% on beta-blockers and ACEi, ARB, or ARNI. In addition, 40% of patients had stage III chronic kidney disease.

There was only a trend towards a reduction in the risk of cardiovascular mortality, but it cannot be excluded that with a longer follow-up period, the reduction in mortality might have become significant.

## Targeting cGMP in HFrEF: from Pathophysiology to Clinical Trials

There are several compensatory mechanisms activated in HF in the attempt to maintain tissue perfusion [29]. These include increasing cardiac output via the Frank–Starling mechanism, increasing LV volume and wall thickness through ventricular remodeling, and the activation of neurohormonal systems [30].

Although initially beneficial in the early stages of HF, all these compensatory mechanisms are ultimately detrimental [31]. All tissue effects of these compensatory mechanisms are mediated by the second messenger cyclic adenylate monophosphate (cAMP) [21]. Natriuretic peptides counteract the effects of the renin-angiotensin-aldosterone and sympathetic nervous systems by promoting sodium and water excretion and inhibiting cardiac and vascular remodeling by activating cGMP signaling. In turn, increasing cGMP can reduce cAMP by activating the phosphodiesterase 2 isoform [32]. Strategies reducing cAMP or potentiating cGMP signaling might then have additive or even synergistic effects, possibly leading to better outcomes.

Among therapies increasing cGMP, only sacubitril/valsartan and vericiguat improved patient outcome over the standard combination of ACEi/ARB, beta-blockers, and MRA in HFrEF [9, 24, 26, 33, 34]. In particular, vericiguat reduced the composite of cardiovascular death and HF hospitalization, a result driven by a reduction in HF hospitalization.

However in the VITALITY trial among patients with HF with preserved left ventricular ejection fraction (HFpEF) and recent decompensation, treatment with vericiguat on top of SOC compared to placebo failed to improve quality of life measured by KCCQ score [35]. This may be attributable to the fact that NO deficiency might not be a key mechanism in the progression of HFpEF, compared to HFrEF [36]. This is also in line with the lack of benefit with oral nitrates and phosphodiesterase-5 inhibitors in HFpEF shown in multiple trials [37–40].

Future research should elucidate whether vericiguat improves patient outcome even when combined with sacubitril/valsartan, SGLT2i, or OM. The response of cardiomyocytes to these novel therapies could be additive or even synergistic and might potentially change the course of HFrEF.

Several authors consider the serial development of treatments that improve morbidity and mortality in patients with HFrEF as one of the great success stories of cardiovascular therapy. They also speculate that the so-called quadruple therapy (i.e., SGLTi on top of SOC triple therapy with ACEi/ARB/ARNI, beta-blockers, and MRA) will be the new SOC in HFrEF [41, 42]. The recently demonstrated benefit and safety of vericiguat in patients with high-risk HF might allow speculation about a quintuple therapy, by adding vericiguat as a new therapeutic tool available for HFrEF treatment. However, which is the best timing, titrating strategy and pharmacological sequencing in future clinical practice still remains to be elucidated.

Additional empirical evidence on the benefits of these new pharmacological agents on top of each other, or comparing them head-to-head are unlikely, since randomizing patients to a control arm of a trial testing a proven drug is hardly justifiable and associated with considerable financial costs. Network meta-analysis facilitates the indirect comparison of multiple combinations of effective treatment options and might provide useful indications on the relative efficacy of the latest therapeutic options [7].

## Conclusions

Recent clinical trials have suggested the benefit and safety of vericiguat in patients with high-risk HF, in particular a lower incidence of death from cardiovascular causes or HF hospitalization. The future positioning of vericiguat therapy needs be better specified especially in the light of recent advances in acute and chronic heart failure therapy.
